# Supplementary material for: Implementation and first report of the Brazilian Kidney Biopsy Registry
Source: PLoS One. 2025 Feb 10;20(2):e0312410. doi: 10.1371/journal.pone.0312410 (PMC11809849; doi:10.1371/journal.pone.0312410)
Supplement: S1 Table — (DOCX) [file pone.0312410.s001.docx]

**Supplemental table 1.** Questionnaire available in the web‑based registry system of the Brazilian Kidney Biopsy Registry Biopsies (BKBR)

**Brazilian Kidney Biopsy Registry – Brazilian Society of Nephrology**

Name (initial letters) ___________ Surname (initial letters )____________

Birth Date ____/_____/____ Age *(platform calculation)* ________

Gender ( ) Male Race ( ) White
 ( ) Female ( ) Black
 ( ) Other ( ) Mixed

( ) Asian

( ) Indigenous

Nephrologist/Doctor: ___________________________________________________

Clinic/hospital: _____________________________

State: *(scrollbar)* ____________ City *(scrollbar)* __________________

Clinical presentation at the time of biopsy (more than one option is possible):

( ) Nephrotic syndrome

( ) Non nephrotic proteinuria

( ) Nephritic syndrome

( ) Hematuria

( ) Rapidly progressive glomerulonephritis

( ) Acute kidney injury

( ) Kidney dysfunction

( ) Others

Diabetes ( ) yes ( ) no

Hypertension ( ) yes ( ) no

Familiy history of kidney disease ( ) yes ( ) no ( ) unknown
Serum Creatinine (closest to the biopsy) ________mg/dL CKD-EPI: *automatically calculated* _________

Others: ___________________________________________________________

Biopsy date : _______/_________/_____________

Nephropathologist: _________________________________________________

Clinic/hospital: ______________________________

State: *(scrollbar)* _____ City *(scrollbar)* ______________________

BIOPSY TISSUE INFORMATION
Number of glomeruli: _______

IFTA (%) _______ ( ) mild ( ) modrate ( ) severe

Results: more than one option is possible *(scrollbar)*

1. ( ) Amyloidosis
    AL amyloidosis ( )
    AA amyloidosis ( )
    Hereditary amyloidosis ( )
2. ( ) Collapsing glomerulopathy
3. ( ) Cryoglobulinemic GN
4. ( ) Fabry´s disease
5. ( ) Minimal change disease
6. ( ) Thin membrane disease
7. ( ) MembranoProliferative GN (MPGN)
    ( ) Immune-complex-mediated GN (IC-MPGN)
    ( ) C3 Glomerulopathy
    DDD ( )
    C3-GN ( )
    ( ) Ig negative / IF negative
8. ( ) Acute Postinfectious GN
9. ( ) Infection related glomerulopathy
    hepatitis B ( )
    hepatitis C ( )
    HIV ( )
    schistosomiasis ( )
    COVID ( )
    syphilis ( )
    leishmania ( )
    others ( )
10. ( ) Monoclonal gammopathy of renal significance
     Monoclonal immunoglobulin deposition disease (MIDD) ( )
     Light Chain Proximal Tubulopathy ( )
     Monoclonal immunoglobulin deposition disease ( )
     Fibrillary Glomerulopathy ( )
     Immunotactoid Glomerulopathy
     Proliferative glomerulonephritis with monoclonal
     IgG deposits (PGNMID) ( )
11. ( ) Focal Segmental Glomerulosclerosis (FSGS)
     NOS ( )
     perihilar ( )
     cellular ( )
     TIP ( )
12. ( ) Hypertension
     ( ) Hypertensive nephrosclerosis
     ( ) Malignant nephrosclerosis
13. ( ) Crescentic glomerulonephritis
     cellular crescents ( )
     fibrocellular crescents ( )
     fibrotic crescents ( )

IF linear ( ) anti-GBM GN
 IF granular ( ) Crescentic GN - Immune Complex
 IF negative ( ) Vasculitis - Pauci Immune GN

1. ( ) Thrombotic microangiopathy
2. ( ) Acute interstitial nephritis
3. ( ) Lupus nephritis
    ( ) II
    ( ) III
    ( ) IV
    ( ) V
    ( ) III ou IV + V
    ( ) VI
    ( ) Podocitopathy
4. ( ) Pregnancy-related nephropathy
5. ( ) Diabetic nephropathy
    Class I ( )
    Class II ( )
    Class III ( )
    Class IV ( )
6. ( ) Membranous nephropathy
    serum ( ) anti-PLA2R POS ( ) anti-PLA2R NEG ( ) Not performed
    Level ______ UI
7. ( ) IgA Nephropathy (IgAN)
    Oxford M 0 ( ) 1 ( )
    Oxford E 0 ( ) 1 ( )
    Oxford S 0 ( ) 1 ( )
    Oxford T 0 ( ) 1 ( ) 2 ( )
    Oxford C 0 ( ) 1 ( ) 2 ( )
8. ( ) Acute Tubular Necrosis
9. ( ) IgA Vasculitis (Henoch-Schönlein Purpura)
10. ( ) Systemic sclerosis
11. ( ) Myeloma cast nephropahty
12. ( ) Alport Syndrome
13. ( ) Chronic unclassified glomerulonephritis
14. ( ) Others

Other comments or diagnoses: ___________________________________
_______________________________________________________________

_______________________________________________________________

( ) **IMUNNOFLUORESCENCE**

( ) **IMUNNOHISTOCHEMISTRY**

IgG ( ) neg ( ) trace ( ) 1+ ( ) 2+ ( ) 3+ Mesagium ( ) Capillary ( )

IgM ( ) neg ( ) trace ( ) 1+ ( ) 2+ ( ) 3+ Mesagium ( ) Capillary ( )

IgA ( ) neg ( ) trace ( ) 1+ ( ) 2+ ( ) 3+ Mesagium ( ) Capillary ( )

C1q ( ) neg ( ) trace ( ) 1+ ( ) 2+ ( ) 3+ Mesagium ( ) Capillary ( )

C3 ( ) neg ( ) trace ( ) 1+ ( ) 2+ ( ) 3+ Mesagium ( ) Capillary ( )

Kappa ( ) neg ( ) trace ( ) 1+ ( ) 2+ ( ) 3+ Mesagium ( ) Capillary ( )

Lambda ( ) neg ( ) trace ( ) 1+ ( ) 2+ ( ) 3+ Mesagium ( ) Capillary ( )

Fibrinogen ( ) neg ( ) trace ( ) 1+ ( ) 2+ ( ) 3+ Mesagium ( ) Capillary ( )

**PLA2R**  ( ) neg ( ) trace ( ) 1+ ( ) 2+ ( ) 3+ Not performed ( )

Medullary tissue only ( )

__________________________________________________________

Others: _______________________________________________________________

**ELECTRON MICOSCOPY** yes( ) no ( )

Electron dense deposits: ______________________________________

Podocyte changes: ___________________________________________

Glomerular basement membrane changes: _____________________________________
Description ______________________________________________________________________________

______________________________________________________________________________
