# Supplementary material for: Implementation and first report of the Brazilian Kidney Biopsy Registry
Source: PLoS One. 2025 Feb 10;20(2):e0312410. doi: 10.1371/journal.pone.0312410 (PMC11809849; doi:10.1371/journal.pone.0312410)
Supplement: S2 Table — A. List of kidney diseases of this cohort grouped into six main categories. B. List of the diseases included in the category “Kidney diseases with Glomerular involvement”. (ZIP) [file pone.0312410.s002.zip › Supplemental Table 2A.docx]

**Supplemental table 2.**

**Supplemental table 2A)** List of kidney diseases of this cohort grouped into six main categories

| **Glomerular Diseases** |
| --- |
| Lupus nephritis |
| IgA Nephropathy (IgAN) |
| Focal segmental glomerulosclerosis (FSGS) |
| Membranous Nephropathy |
| Minimal Change Disease |
| Vasculitis - Pauci Immune GN |
| Collapsing Glomerulopathy |
| Immune-complex-mediated GN (IC-MPGN) |
| TMA |
| Acute Post-Infectious GN |
| C3-Glomerulopathy |
| Crescentic GN - Immune Complex Mediated |
| HIV-Related Nephropathy |
| Crescentic GN - anti-GBM GN |
| Mesangial Proliferative GN |
| Cryoglobulinemic GN |
|  |
| **Diabetic nephropathy** |
|  |
| **Tubulo-Interstitial Diseases** |
| Acute Interstitial Nephritis |
| Acute Tubular Necrosis (ATN) |
| IgG4-Related Kidney Disease |
|  |
| **Monoclonal Gammopathy** |
| Amyloidosis |
| Cast Nephropathy |
| Monoclonal immunoglobulin deposition disease (MIDD) |
| Light Chain Proximal Tubulopathy |
| Other Gammopathies |
| Fibrillary Glomerulopathy |
| Immunotactoid Glomerulopathy |
| Proliferative glomerulonephritis with monoclonal IgG deposits (PGNMID) |
|  |
| **Vascular Diseases** |
| Hypertensive Nephrosclerosis |
| Systemic Sclerosis |
|  |
| **Inherited Kidney Diseases** |
| Collagen IV disorders |
| LCAT Deficiency |
| Fabry´s Disease |
|  |
| **Miscellaneous** |
|  |
| **Unclassifiable** |
