# Supplementary material for: Implementation and first report of the Brazilian Kidney Biopsy Registry
Source: PLoS One. 2025 Feb 10;20(2):e0312410. doi: 10.1371/journal.pone.0312410 (PMC11809849; doi:10.1371/journal.pone.0312410)
Supplement: S2 Table — A. List of kidney diseases of this cohort grouped into six main categories. B. List of the diseases included in the category “Kidney diseases with Glomerular involvement”. (ZIP) [file pone.0312410.s002.zip › Supplemental Table 2B.docx]

**Supplemental table 2.**

**Supplemental table 2B)** List of the diseases included in the category “Kidney diseases with Glomerular involvement”

| Lupus nephritis |
| --- |
| IgAN |
| FSGS |
| Membranous Nephropathy |
| Minimal Change Disease |
| Vasculitis - Pauci Immune GN |
| Collapsing Glomerulopathy |
| Immune-complex-mediated GN (IC-MPGN) |
| TMA |
| Acute Post-Infectious GN |
| C3-Glomerulopathy |
| Crescentic GN - Immune Complex Mediated |
| HIV-Related Nephropathy |
| Crescentic GN - anti-GBM GN |
| Mesangial Proliferative GN |
| Cryoglobulinemic GN |
| Diabetic Nephropathy |
| Amyloidosis |
| Monoclonal immunoglobulin deposition disease (MIDD) |
| Proliferative glomerulonephritis with monoclonal IgG deposits (PGNMID) |
| Immunotactoid Glomerulopathy |
| Fibrillary Glomerulopathy |
| Collagen IV disorders |
| LCAT Deficiency |
| Fabry Disease |
