# Supplementary material for: Implementation and first report of the Brazilian Kidney Biopsy Registry
Source: PLoS One. 2025 Feb 10;20(2):e0312410. doi: 10.1371/journal.pone.0312410 (PMC11809849; doi:10.1371/journal.pone.0312410)
Supplement: S3 Table — (DOCX) [file pone.0312410.s003.docx]

|  | **N (%)** | **Age**  (at the Bx) | **Gender** (Male) | **Serum creatinine**  (mg/dL) | **Nephrotic Syndrome** | **NonNephrotic proteinuria** | **Hematuria** | **Renal Dysfunction** | **Diabetes** | **Hypertension** |
| --- | --- | --- | --- | --- | --- | --- | --- | --- | --- | --- |
| **Membranous Nephropathy** | 21 (16.4%) | 72.2±6.5 | 15 (71.4%) | 2.3±2.1 | 17 (81%) | 3 (14.3%) | 1 (4.8%) | 11 (52.4%) | 4 (19%) | 15 (71.4%) |
| **Diabetic Nephropathy** | 15 (11.7%) | 70.2±5.6 | 8 (53.3%) | 2.8±1.6 | 8 (53.3%) | 2 (13.3%) | 1 (6.7%) | 10 (66.7%) | 15 (100%) | 12 (80%) |
| **FSGS** | 11 (8.6%) | 70.5±4.8 | 6 (54.5%) | 2.0±0.9 | 4 (36.4%) | 7 (63.6%) | 1 (9.1%) | 7 (63.6%) | 0 (0%) | 10 (72.7%) |
| **Acute Interstitial Nephritis** | 11 (8.6%) | 72.9±8 | 7 (63.6%) | 3.4±2.4 | 0 (0%) | 3 (27.3%) | 1 (9.1%) | 10 (90.9%) | 3 (27.3%) | 8 (90.9%) |
| **Monoclonal Gammopathy** | 10 (7.8%) | 75.4±4.4 | 5 (50.0%) | 5.2±4.4 | 5 (50.0%) | 0 (0%) | 1 (10%) | 8 (80%) | 3 (30%) | 7 (70%) |
| **Minimal Change Disease** | 8 (6.3%) | 73.8±6.5 | 5 (83.3%) | 2.1±1.0 | 8 (100%) | 0 (0%) | 1 (12.5%) | 6 (75.0%) | 3 (37.5%) | 7 (87.5%) |
| **IgAN** | 8 (6.3%) | 71±6.9 | 6 (75%) | 1.9±0.9 | 2 (25%) | 5 (62.5%) | 5 (62.5%) | 4 (50.0%) | 2 (25.0%) | 7 (87.5%) |
| **Vasculitis - Pauci Immune GN** | 7 (5.5 %) | 71.9±9.7 | 2 (28.6%) | 5.3±3.2 | 1 (14.3%) | 1 (14.3%) | 1 (14.3%) | 1 (14.3%) | 0 (0%) | 3 (42.9%) |
| **Hypertensive Nephrosclerosis** | 6 (4.7%) | 73.2±6.1 | 5 (83.3%) | 2.2±1.0 | 0 (0%) | 5 (83.3%) | 0 (0%) | 4 (66.7%) | 2 (33.3%) | 6 (100%) |
| **Unclassifiable** | 6 (4.7%) | 72.0±5.4 | 6 (100%) | 5.3±3.9 | 2 (33.3%) | 2 ( 33.3%) | 1 (16.7) | 4 (66.7%) | 2 (33.3%) | 4 (66.7%) |
| **TMA** | 5 (3.9 %) | 70.6±3.0 | 3 (60%) | 3.3±1.7 | 1 (20%) | 0 (0%) | 2 (40%) | 4 (80.0%) | 2 (40.0%) | 2 (40%) |
| **Amyloidosis** | 4 (3.1%) | 71.8±4.0 | 2 (50.0%) | 1.9±1.2 | 4 (100%) | 0 (0%) | 1 (25%) | 1 (25%) | 0 (0%) | 2 (50%) |
| **IC-MPGN** | 4 (3.1 %) | 71.3±3.9 | 1 (25%) | 2.0±0.7 | 3 (75%) | 1 (25%) | 3 (75%) | 3 (75%) | 1 (25%) | 4 (100%) |
| **Lupus nephritis** | 3 (2.3%) | 67.7±0.6 | 1 (33.3%) | 1.9±0.7 | 0 (0%) | 1 (33.3%) | 1 (33.3%) | 1 (33.3%) | 1 (33.3%) | 2 (66.7%) |
| **Crescentic - IC Mediated** | 2 (1.6%) | 72.0±2.8 | 1 (50%) | 3.0±0.5 | 0 (0%) | 1 (50%) | 1 (50%) | 1 (50%) | 0 (0%) | 0 (0%) |
| **Collapsing Glomerulopathy** | 2 (1.6%) | 66.5±2.1 | 2 (100%) | 7.0±0.4 | 1 ( 50%) | 0 (0%) | 0 (0%) | 1 (50%) | 2 (100%) | 2 (100%) |
| **HIV-Related Nephropathy** | 2 (1.6%) | 68.0±1.4 | 2 ( 100%) | 5.0±1.4 | 0 (0%) | 1 (50%) | 0 (0%) | 1 (50%) | 0 (0%) | 0 (0%) |
| **C3 Glomerulopathy** | 1 (0.8%) | 76 | 1 | 1.9 | 0 | 1 | 0 | 1 | 1 | 1 |
| **Mesangial Proliferative GN** | 1 (0.8%) | 70 | 0 | 0.9 | 0 | 0 | 0 | 0 | 0 | 0 |
| **Cryoglobulinemia** | 1 (0.8%) | 72 | 0 | 1.15 | 0 | 1 | 1 | 0 | 0 | 0 |
| **Overall** | **128 (100%)** | **71.8±5.9** | **76 (59.4%)** | **3.0±2.5** | **56 (43.8%)** | **34 (26.6%)** | **22 (17.2%)** | **76 (59.4%)** | **41 (32%)** | **92 (71.9%)** |

**Supplemental table 3.** Characteristics of kidney diseases in biopsies in elderly patients
