# Supplementary material for: Implementation and first report of the Brazilian Kidney Biopsy Registry
Source: PLoS One. 2025 Feb 10;20(2):e0312410. doi: 10.1371/journal.pone.0312410 (PMC11809849; doi:10.1371/journal.pone.0312410)
Supplement: S4 Table — (DOCX) [file pone.0312410.s004.docx]

**Supplemental table 4.** Demographic, clinical and laboratory characteristics of patients with diabetes mellitus

|  | **Patients with Diabetes Mellitus** | |
| --- | --- | --- |
|  | **Biopsy-proven**  **Diabetic Nephropathy** | **No evidence  of Diabetic Nephropathy** |
| **Number (%)** | 81 (55.9%) | 64 (44.1%) |
| **Age** (y) | 51.4 ± 14.0 | 57.2 ± 13.7 |
| **Gender** |  |  |
| Male | 41 (50.6%) | 29 (47.5%) |
| Female | 40 (49.4%) | 32 (52.5%) |
| **Race** |  |  |
| White | 37 (45.7%) | 35 (54.7%) |
| Black | 5 (6.2%) | 5 (7.8%) |
| Mulatto (Mixed) | 38 (46.9%) | 22 (34.4%) |
| Yellow (Asian) | 1 (1.2%) | 2 (3.1%) |
| Indigenous | 0 (0%) | 0 (0%) |
| **Clinical characteristics** |  |  |
| Nephrotic syndrome | 49 (60.5%) | 27 (42.2%) |
| Non-nephrotic proteinuria | 19 (23.52%) | 15 (28.6%) |
| Hematuria | 13 (16.0%) | 8 (21.9%) |
| RPGN | 3 (3.7%) | 0 (3.1%) |
| Kidney dysfunction | 53 (65.4%) | 41 (62.5%) |
| Hypertension | 61 (75.3%) | 53 (82.8%) |
| Diabetes mellitus | 81 (100%) | 64 (100%) |
| **Serum creatinine**  at kidney Bx (mg/dL) | 3.14 ± 2.9 | 3.14 ± 2.4 |
