# Supplementary material for: Implementation and first report of the Brazilian Kidney Biopsy Registry
Source: PLoS One. 2025 Feb 10;20(2):e0312410. doi: 10.1371/journal.pone.0312410 (PMC11809849; doi:10.1371/journal.pone.0312410)
Supplement: S5 Table — (DOCX) [file pone.0312410.s005.docx]

**Supplemental table 5. List of collaborators**

Adriana Maria Kakehasi, Hospital das Clínicas - UFMG, Belo Horizontes, MG; Adriana Vilarinho de Oliveira, Hospital Universitario Walter Cantidio - UFC, Fortaleza, CE; Akiyoshi Ugino, Hospital do Rim de Araçatuba, Araçatuba, SP; Alcino Reis Mendes, Hospital São Domingos, Uberaba, MG; Alessandra Campos De Oliveira, Mediax, Recife, PE; Alexandre Carvalho Pinto Coelho, Hospital Unimed, Belo Horizonte, MG; Aline Benitez Ortega De Menezes, Instituto do Rim, Presidente Prudente, SP; Amanda Karollyne Da Silva Carvalhar, Hospital Das Clinicas – UFPE, Recife, PE; Américo Lourenço Cuvello-Neto, Hospital Alemão Oswaldo Cruz, São Paulo, SP; Ana Carolina Salim Casseb, Senerp - Serviço De Nefrologia De Ribeirão Preto, Ribeirão Preto, SP; Ana Flavia Vieira Ferreira, Hospital Universitário - Faculdade de Ciências Medicas, Belo Horizonte, MG; Ana Paula Silva Gueiros, Hospital das Clínicas - UFPE, Recife, PE; Anaiara Lucena Queiroz, Hospital Universitario Walter Cantidio - UFC, Fortaleza, CE; André Costa Teixeira, Laboratório Argos, Fortaleza, CE; Andrea Callou De Araújo Pereira Machado, Clínica Senhor do Bonfim, Feira de Santana, BA; Angelo Eduardo De Faria Paschoalini, Hospital Unimed, Belo Horizonte, MG; Anna Claudia Borges Meireles, Hospital Das Clínicas Da Faculdade De Medicina De Ribeirão Preto, Ribeirão Preto, SP; Aparecida Paula Gondim, Santa Casa De Votuporanga, Votuporanga, SP; Ariel Augusto De Britto Rosa, Hospital Universitário - Faculdade de Ciências Medicas, Belo Horizonte, MG; Aysla Santana, Hospital Das Clínicas Da Faculdade De Medicina De Ribeirão Preto, Ribeirão Preto, SP; Bianca Casarotto Lima Faria, Hospital Regional De Sobradinho, Brasília, DF; Bruna Rodrigues Moreira, Hosp Felicio Rocho, Belo Horizonte, MG; Bruno Alves Silva, Hospital Regional De Presidente Prudente, Presidente Prudente, SP; Bruno Dorilleo Vieira, Santa Casa, Barretos, SP; Bruno Fontes Lichtenfels, Hospital Sao Jose, Criciúma, SC; Carla Luanda Pereira Dos Santos, Hospital Vida, Maceió, AL; Carlos Eduardo Mendonça Rocha, Santa Casa de Andradina, Andradina, SP; Cassiano Augusto Braga Silva, Clínica Senhor do Bonfim, Feira de Santana, BA;Celso Fianco, Hospital São Lucas, Patos de Minas, MG; Cesar Oliveira, Iune Clínicas, Feira de Santana, BA; Cezar Brasileiro Bezerra Pereira, Consultório Privado, Brasília, DF; Claudia Ribeiro, Santa Casa de Belo Horizonte, Belo Horizonte, MG; Daniela De Almeida Alves, Hospital Santa Rita, Belo Horizonte, MG; Davi Ernane Oliveira Martins, Hospital Das Clinicas - UFPE, Recife, PE; Dênio Braga De Souza, Hospital Madre Teresa, Belo Horizonte, MG; Dhiego Lang Campi, SOS Rim, Porto Velho, RO; Domingos Chula, Hospital das Clínicas – UFPR e Fundação Pró-Renal, Curitiba, PR; Dulce Maria Sousa Barreto, Hospital Geral De Fortaleza, Fortaleza, CE; Eberaldo Severiano Domingos, Hospital Márcio Cunha, Ipatinga, MG; Edvaldo Costa Neto, Clinefro, Senhor do Bonfim, BA. Elida Moura, SOS Rim, Porto Velho, RO; Eline Nogueira, Clínica Dali, Teófilo Otoni, MG; Emerson Quintino De Lima, Hospital De Base De São José Do Rio Preto – FAMERP, São José do Rio Preto, SP; Epitacio Rafael, Hospital Ana Nery, Salvador, BA; Evaldo Garcia Terra, Santa Casa, Fernandópolis, SP; Fabiano Bichuette Custódio, Hospital De Clinicas – UFTM, Uberaba, MG; Fabiano Fernandes Silva, Hospital Evangélico, Belo Horizonte, MG; Fabio Henrique De Sousa Teixeira, Hospital Evangelico, Belo Horizonte, MG, Fabio Reis, Clinica De Doencas Renais De Brasilia, Brasília, DF; Fábio T. Monari, Humanus, Lins, SP; Felipe Alves Campos, Santa Casa De BH, Belo Horizonte, MG; Fellipe Goncalves Declie Fagioli, NefroClínicas, Belo Horizonte, MG; Fernanda Cristina Camelo Sanchez, Hospital De Base De São José Do Rio Preto – FAMERP, São José do Rio Preto, SP; Fernanda Quadros Mendonca Marques, Hospital Do Rim, Montes Claros, MG; Fernando Das Merces De Lucas Junior, NefroClínicas, Belo Horizonte, MG; Francisco José Veríssimo Veronese, Hospital De Clínicas De Porto Alegre, Porto Alegre, RS; Frederico Castelo Branco Cavalcanti, Real Hospital Portugues, Recife, PE; Gabriela Lacreta Leone Moreira, Soc. Hospitalar Angelina Caron, Campina Grande do Sul, PR; Geraldo Sergio Goncalves Meira, Hospital Do Rim, Montes Claros, MG; Gianna Mastroianni Kirsztajn, Disciplina De Nefrologia Unifesp, São Paulo, SP; Gildasio Da Silva Rocha Filho, Clínica Senhor do Bonfim, Feira de Santana, BA;; Gisele Vajgel, Hospital Das Clinicas – UFPE, Recife, PE; Guilherme Parise Santa Catharina, Hospital Beneficencia Portuguesa, São Paulo, SP; Gustavo Gomes Thomé, Hospital De Clínicas De Porto Alegre, Porto Alegre, RS; Gustavo Guerra Jacob, Hospital Biocor, Nova Lima, MG; Gustavo Navarro Betonico, Faculdade de Medicina de Adamantina, Adamantina, SP; Gustavo Vasconcelos De Araújo, Tagu Clínica Médica, Catalão, GO; Hebert Henrique Capuci, Instituto De Hemodiálise De Uberaba, Uberaba, MG; Heitor Alves Rocha, Santa Casa De Goiania, Goiânia, GO; Helen Souto Siqueira Cardoso, Hospital Anchieta, Brasília, DF; Heleno Batista De Oliveira, Clinica De Tratamento De Doenças Renais Eireli, Uberlândia, MG; Horacio José Ramalho, Hospital De Base De São José Do Rio Preto – FAMERP, São José do Rio Preto, SP; Humberto Caldeira Brant Junior, Hospital Nossa Senhora De Fátima, Patos de Minas, MG; Ida Maria Maximina Fernandes Charpiot, Hospital De Base De São José Do Rio Preto – FAMERP, São José do Rio Preto, SP; Igor Costa Almeida, Hospital Regional de Presidente Prudente, Presidente Prudente, SP; Inalda Facincani, Hospital Das Clínicas Da Faculdade De Medicina De Ribeirão Preto, Ribeirão Preto, SP; Isabela Bauti Pinto; Hospital Das Clinicas - UFMG, Belo Horizonte, MG; Isabela Lage Pimenta, Hospital Evangelico, Belo Horizonte, MG; Italo Magalhaes Gusmao; Santa Casa de Belo Horizonte; Belo Horizonte; MG; Ivete De Souza Barbosa Nunes, Clínica Privada, Monte do Carmo, TO; Jassonio Mendonca, Hospital Regional Do Gama, Brasília, DF; João Fernando Picollo de Oliveira, Hospital De Base De São José Do Rio Preto – FAMERP, São José do Rio Preto, SP; José Augusto Paes Junior, Hospital Lifecenter, Belo Horizonte, MG; Jose Fernando Stocco Guilhen, Instituto do Rim, Marília, SP; Juliana Ferreira Bezerra De Azevedo, Hospital Universitario - UFJF, Juiz de Fora, MG; Juliana Gomes Bordon, Hospital Municipal De Uberlândia, Uberaba, MG; Juliana Maciel De Assis, Hospital Municipal De Uberlândia, Uberlândia, MG; Julio Cesar Soares Barreto, TRS Nefrologia e Hemodiálise, Goiania, GO; Karolina Honorato, Santa Casa de Barretos, Barretos, SP; Kátia Alves Ramos, Hospital Vera Cruz, Patos de Minas, MG; Kellen Micheline Alves Henrique Costa, Hospital Universitário Onofre Lopes, Natal, RN; Kemila Martins Chaves, Nefron Serviços Médicos de Nefrologia, Belo Horizonte, MG; Kildere Moura, Clinica de Nefrologia de Euclides da Cunha BA; Laís Lopes M Cunha, Clínica Senhor do Bonfim, Feira de Santana, BA; Larissa Amorim Miranda, Hospital Das Clínicas Da Faculdade De Medicina De Ribeirão Preto, Ribeirão Preto, SP; Leandro Santos Da Silva, Santa Casa de Belo Horizonte, Belo Horizonte, MG; Leandro Vasconcelos, Hospital Santa Joana, Recife, PE; Leonardo Andrade Pacini, Hospital Das Clinicas - UFMG, Belo Horizonte, MG; Leonardo Verona, Hospital Beneficência Portuguesa, São José do Rio Preto, SP; Leticia Gouthier Bicalho, Hospital Das Clinicas - UFMG, Belo Horizonte, MG; Liliany Repizo Nitani, Unna Medicina Integrada, Fernandópolis, SP; Lívia Barreira Cavalcante, Hospital Alemão Oswaldo Cruz, São Paulo, SP; Loise Lourraine Canabrava Turra, Hospital Nossa Senhora de Lourdes, Nova Lima, MG; Lorena Fernandes Maia, Hospital Alberto Cavalcanti/FHEMIG, Belo Horizonte, MG, Luana Cicilia Sousa Da Silva, Hospital Santa Lucia, Brasília, DF; Lucas Nascimento Diniz Teixeira, Hospital Das Clinicas – UFPE, Recife, PE; Luciana Kelly de Camargos Batista, Hospital Regional de Presidente Prudente, Presidente Prudente, SP; Lucila M Valente, Hospital Das Clinicas – UFPE, Recife, PE; Luis Gustavo De Freitas Trindade, Hospital Universitário da Faculdade de Ciências Médicas, Belo Horizonte, MG; Luis Sette, Mediax, Recife, PE; Luiz Flavio Couto Giordano, Hospital Mater Dei, Belo Horizonte, MG; Maice Vieira Marinho, Santa Casa De Belo Horizonte, Belo Horizonte, MG; Maira Campos Zoccarato Arouca, Hospital Unimed, Belo Horizonte, MG; Marcela Pagianotto Bidoia, Hospital De Base De São José Do Rio Preto – FAMERP, São José do Rio Preto, SP; Marcia Ramalho Santos, Hospital Philadelfia, Teófilo Otoni, MG; Marcus Vinícius Padua Neto, Hospital De Clínicas - UFU, Uberlândia, MG; Maria Alice Sperto Ferreira Baptista, Hospital De Base De São José Do Rio Preto – FAMERP, São José do Rio Preto, SP; Maria Almerinda Ribeiro Alves, Hospital De Clínicas Da Unicamp, Campinas, SP; Maria Cecilia Sales Mendes Prates, Santa Casa de Diamantina, Diamantina, MG; Maria Eugenia Abreu Moreira, ClinOrto, Belo Horizonte, MG; Maria Luiza Garcia De Magalhaes Gualberto, FAMINAS, Belo Horizonte, MG; Mariana Salomão Braga, Hospital das Clínicas da UFTM, Uberaba, MG; Marlinson Borges Rosario, NefroClínicas, Belo Horizonte, MG; Mary Carla Estevez Diz, Hospital Do Servidor Público Minicipal, São Paulo, SP; Maurício Soledade, Clínica de Nefrologia de Serrinha, BA; Miguel Gomes Dias, Hospital São Lucas, Governador Valadares, MG, Murillo Conde Nascimento, Santa Casa De Misericordia, Presidente Prudente, SP; Natalia Kenia Paiva Souza, Clínica Privada, Itauna, MG; Nathalia Adila Alves Machado, Santa Casa De Belo Horizonte, Belo Horizonte, MG; Nathalia Paschoalin Carvalho, Clínica Senhor do Bonfim, Feira de Santana, BA;; Neide Missae Murai, Hospital De Base De São José Do Rio Preto – FAMERP, São José do Rio Preto, SP; Osvaldo Merege Vieira Neto, Senerp - Serviço De Nefrologia De Ribeirão Preto; Ribeirão Preto, SP; Otavio Augusto de Oliveira Machado, Hospital Universitario – Faculdade de Ciências Medicas, Belo Horizonte, MG; Patricia Malafronte, Santa Casa De Misericórdia De São Paulo, São Paulo, SP; Paulo Ricardo Teixeira, Clínica Senhor do Bonfim, Feira de Santana, BA;; Paulo Salgado Rabelo, Santa Casa De BH, Belo Horizonte, MG; Priscila Lima Tavares, Clínica Privada, Belo Horizonte, MG; Priscilla Tolentino Barbosa Fernandes, Hospital Dilson Godinho, Salinas, MG; Rafael Fernandes Vanderlei Vasco, Hospital Memorial Arthur Ramos, Maceió, AL; Rafael Francisco Ferreira De Souza, HC-UFMG, Belo Horizonte, MG; Rafael Lage Madeira, Hospital Felicio Rocho, Belo Horizonte, MG; Ricardo Borges E Silva, Clinica Do Rim Do Alto Paranaiba, Patos de Minas, MG; Roberto Kenedy Gomes De Oliveira, Instituto de Nefrologia de Pará de Minas, Pará de Minas, MG; Rodolfo Antonio Silva Nascimento; Instituto De Urologia De Itabuna, BA; Rodrigo Alfredo Vivanco Vergara, Clinica De Doencas Renais De Brasilia, Brasília, DF; Rodrigo Braz Santos, CENED Clinica do Rim, Dourados, MS; Rodrigo Gomes Souto, CENED Clinica do Rim, Dourados, MS; Rodrigo Hagemann, HC-UFPR, Curitiba, PR; Rosilane Ferreira Manfrim, Hospital São Marcos, Itumbiara-GO; Rubia Bethania Biela Boaretto, Nefro Saúde, Cascavel, PR; sÉRGIO Ossamu Ioshii, HC-UFPR, Curitiba, PR; Sérgio Tahan Vilarinho, Hospital N Sra D Abadia Ltda, Ituiutaba, MG; Stanley De Almeida Araújo, Instituto De Nefropatologia, Belo Horizonte, MG; Stenio Barbosa De Freitas, Hosp São João De Deus, Divinópolis, MG; Tatiana Vieira Carneiro, Hospital Universitário Clemente de Faria, Montes Claros, MG; Thais Paiva Torres, Hospital Governador Israel Pinheiro, Belo Horizonte, MG; Thatyana Wendhausen, Hospital São Jose, Criciúma, SC; Theo Rodrigues Costa, Hospital Do Rim De Goiania, Goiânia, GO; Thiago De Castro Santos, Hospital Nossa Senhora Das Gracas, Sete Lagoas, MG; Thiago Reis, Clinica De Doencas Renais De Brasília e Hospital Alvorada, Brasília, DF; Tomas Ribeiro Carvalho, Santa Casa De Passos, Passos, MG; Túlio Coelho Carvalho Clínica Senhor do Bonfim, Feira de Santana, BA; Vanessa Carolina Barros Silva, Hospital Regional de Presidente Prudente, Presidente Prudente, SP; Vanessa Dos Santos Silva, HCFMB-Unesp; Botucatu, SP; Vitor Francisco Souza Pereira Clínica Senhor do Bonfim, Feira de Santana, BA; Viviane Alves Leite, Hospital do Rim de Goiania, Goiânia, GO; Werton De Medeiros Roque Filho, AMIP - Assistência Médica Infantil Da Paraíba, João Pessoa, PB; Washington Luiz Conrado dos Santos, Centro de Pesquisa Gonçalo Moniz, Fundação Oswaldo Cruz, Salvador Bahia; Willians Vinicius Dutra Rodrigues, Hospital Risoleta Tolentino Neves, Belo Horizonte, MG; Wyara Gomes De Aniceto Clínica Senhor do Bonfim, Feira de Santana, BA.
